# Incidence and management of infections in patients with acute leukemia following chemotherapy in general wards

**DOI:** 10.3332/ecancer.2013.310

**Published:** 2013-04-22

**Authors:** Sasmita Biswal, Chaitali Godnaik

**Affiliations:** SCB Medical College & Hospital, Cuttack, Odisha, India

**Keywords:** acute leukemia, febrile neutropenia, infection, general ward setting, mortality

## Abstract

We hypothesise that treating patients with acute leukaemia in general wards, with proper hygienic and sanitary practices, would result in the minimum utilisation of resources as compared with the corresponding patients receiving ICU support. For this study, the acute leukaemia patients on induction chemotherapy were kept in general wards and observed for the incidence of neutropenia, resultant neutropenic febriles, the causative organism, and the effect of empirical antimicrobial treatment protocol on the outcome of such infections. Prophylactic anti-fungal therapy and cotrimoxazole therapy improved the outcome of infections. The therapy of neutropenic fever and infections must be adapted according to the risk factors and should include early empiric antifungal therapy. It was observed that the treatment of such patients in general wards could be managed effectively, with the added advantage of optimum utilisation of resources and in a patient-friendly environment, at a reasonable cost to the patients.

## Introduction

Acute leukaemia is the most common malignancy in India whose treatment, in developed countries, is highly specialised. Although there are several oncology institutes as well as many regional cancer centres in India, very few patients have access to such specialised treatment. The reasons for this are low socioeconomic status, increased distance to the treatment centre, and the financial burden of chemotherapy and supportive treatment.

During recent decades, the advent of novel chemotherapeutic agents and intense chemotherapeutic regimens as front line therapy for leukaemia have dramatically improved the outlook of this disease, as more and more patients are achieving clinical cure. But such intense chemotherapy with cytotoxic drugs usually results in myelosuppression and a resultant high risk of neutropenia (absolute neutrophil count < 0.5 × 10^9^/L) [1], which has a detrimental effect on the integrity of the normal human skin and mucosa, which are at great risk of invasive infection due to the colonising bacteria, virus and fungi as neutropenia impairs the phagocytic activity of the neutrophils [2]. Immunocompromised patients, such as those diagnosed with malignancies and receiving chemotherapy, are at even higher risk of neutropenia and resultant infections [3]. These usually arise during the first course of induction chemotherapy, and are directly proportional to the duration and severity of the neutropenia. Such complications require early and prompt initiation of antimicrobial therapy [4, 5] and greater demand for supportive therapy in an ICU setting [6].

Thus, patients have traditionally been hospitalised in intensive care units (ICUs) for the duration of chemotherapy or until count recovery from the neutropenic febrile episodes (NFEs). However, such management of patients is driven by high healthcare costs, increased demand for existing inpatient resources, and the patient’s wish to spend the least amount of time in an alien ICU setting [7].

Driven by healthcare costs and increased demand for existing inpatient resources, outpatient care of patients with malignancies has become increasingly common at present [8]. Based on such reports, we hypothesised that treating patients with acute leukaemia in general wards with proper hygienic and sanitary practices would be the most cost-effective approach, result in minimum utilisation of resources, and may, in addition, assist many patients in completing their course of chemotherapy per their specific schedule, as compared with the corresponding patients receiving ICU support. Thus, it could help in limiting the possible futile use of more and more limited resources.

Due to an increasing number of patients waiting to be admitted to a restricted number of ICU beds, which can create the possibility of detrimental delays between chemotherapy cycles, the Department of Clinical Hematology of our tertiary care hospital has moved to the possibility of the management of such leukaemic patients in the inpatient setting. In our setup, such leukaemic patients are kept in general wards along with patients with various other ailments, with no changes being made to the actual regimen of administered chemotherapy. Since isolation of the leukaemic patient was not feasible in our setup, the standard hygienic practices adopted in this ward were the use of face masks by patients and visitors, strict avoidance of hand shaking, hand disinfection before entering and after leaving any patient room, and limited access of visitors. With regard to diet, it has always been hypothesised that a diet for neutropenic patients should be germfree so as to reduce the number of pathogens entering the gastrointestinal tract by providing them with an exclusive germ-free diet. However, in a randomised trial, the National Cancer Institute has shown that a germ-free diet had little advantage over a cooked-food diet [9]. Furthermore, the cooked-food diet was more acceptable to the patients, while the patients who adhered to the germ-free diet for more than four to six weeks often became frustrated with the food selection. As this evidence suggested a controversial role of diet in the risk of infection in patients with neutropenia, all of our study’s patients received the normal cooked ward diet within a decontaminated environment.

Neutropenia alters the host’s inflammatory response, thereby making the resultant infections difficult to detect; also, bacteriological confirmation of infection is usually difficult to obtain, while negative cultures do not exclude the presence of infection. Hence, several markers are used as indicators of infection. C-reactive protein (CRP) is one such marker whose elevated serum levels are seen in acute Gram-positive, Gram-negative, and fungal infections [10]. Thus, a single CRP measurement is reasonably useful in the diagnosis of sepsis, which suggests that infection should always be suspected if there is a steady increase in CRP levels over a period of two to three days, and in the absence of any intervention [11]. CRP has traditionally been used to evaluate the severity of infection and also the response to antimicrobial therapy in patients with febrile neutropenia, even though its predictive value is controversial [12]. Therefore, CRP was one of the infection markers chosen for this study protocol, as it is easily available and familiar as a reference marker for sepsis.

This study was done with the following main objectives:

1. To determine the epidemiological features, microbiological aetiology, and outcome of infections (bacterial and fungal) in acute leukaemic patients on induction chemotherapy in general wards during the neutropenic period.
2. To see whether proper hygienic and scientific treatment of acute leukaemic patients in a non-ICU setup in general wards can be comparable with that of an ICU setup.
3. To find a correlation between the serum levels of CRP and impending infections in such neutropenic patients.

Duration of febrile neutropenia, the time span between chemotherapy and onset of febrile neutropenia, and the efficacy of prophylactic antimicrobials administered to patients were also investigated and documented.

## Material and methods

### Study design 

This study was undertaken in the Post Graduate Department of Pharmacology, and in the Department of Clinical Hematology of our tertiary care teaching hospital.

All cases of acute leukaemia admitted from April 2012 to September 2012 constituted the study material. This was a longitudinal prospective observational study, which was conducted among 120 patients, which included both males and females from two to 50 years of age. All patients diagnosed with acute leukaemia and receiving intensive induction chemotherapy were eligible for the study protocol.

## Eligibility criteria 

### Inclusion criteria

1. All cases of acute leukaemia, acute lymphatic leukaemia (ALL), and acute myeloid leukaemia (AML), from the age group of two to 50 years old. Cases of acute promyelocytic leukaemia were not included in the study.
2. Patients with a confirmed diagnosis of acute leukaemia by haematological, cytochemical, and by flow cytometric immunotyping.
3. Willingness to participate in the study after informed written consent.

### Exclusion criteria

1. Patients less than two or >50 years of age.
2. Patients with comorbid conditions such as diabetes, hypertension, chronic renal failure or cardiac diseases.
3. Unwillingness to participate in the study.
4. Patients with a history of bone marrow transplant.
5. Patients with evidence of clinical infections prior to chemotherapy.
6. Cases of acute promyelocytic leukaemia.

The study protocol was approved by the Institutional Ethical Committee prior to the commencement of the study, and only study participants who gave their informed written consent were enrolled in the study.

## Treatment protocol

All the diagnosed cases of ALL patients received the MCP-841 protocol, a standard protocol that is followed in our institution where the treatment comprised induction with four drugs using vincristine, L-asparaginase, daunomycin, and prednisolone, and consolidation with cyclophosphamide and cytosine arabinoside.

The AML patients received the ‘3+7’ (Dauno + Ara-C) regimen, followed by a monthly high dose of Ara-C (3 g/m^2^) twice daily on alternate days for six doses. The ‘3+7’ Dauno + Ara-C therapy was comprised of daunomycin (60 mg/m^2^/day) on days one through three and cytosine arabinoside (200 mg/m^2^/day) on days one through seven.

Supportive therapy such as blood and its components, 5-HT3 antagonists for nausea and vomiting, and chlorhexidine mouthwash after each feed for good oral hygiene were administered as and when required.

A patient was defined as being neutropenic when the blood ANC count was less than 0.5 × 10^9^/L. Since patients with leukaemia have always been considered to be high risk with febrile neutropenia, inpatient prophylactic antimicrobial therapy was attempted in all the study patients. Thus, starting on the day 1 of chemotherapy, all patients received prophylactic antimicrobial therapy with fluconazole 200–400 mg orally once a day for adequate coverage against fungi, along with cotrimoxazole, administered for two or three days per week as prophylaxis against *Pneumocystis jeroveci *infections. Antibiotic prophylaxis for bacterial infections was not attempted due to reports of colonisation with resistant bacteria by such protocols [13]. These prophylactic antimicrobials were stopped when the ANC reached the normal limits of 0.5 × 10^9^/L. Patients were transfused with whole blood, packed red cell units, fresh frozen plasma, and platelets as and when required.

## Haematology and microbiology investigations

Peripheral blood morphology along with bone marrow cytology, morphology, immunophenotyping, and cytogenetics were employed for confirming the specific diagnosis of leukaemia. Other investigations done were haemogram, peripheral smear, bone marrow, biochemical renal and liver function tests, urine analysis, and appropriate cultures on suspicion of infection.

Blood culture was carried out aseptically, where five to seven ml of blood were obtained and directly added to brain heart infusion (BHI) broth at the onset of fever, and incubated at 37°C for seven days. The bottles were checked visually and shaken manually on a daily basis, and regular subcultures were done from these bottles into Mac Conkey and blood agar plates. If no signs of bacteria or yeasts were seen, incubation of the bottles was continued. The subcultures were incubated for up to two days and isolates recovered from positive cultures were identified by Gram stain and standard biochemical tests. A final negative blood culture was reported if there was no growth after 10 days of culture. Antimicrobial susceptibility testing was done using the Kirby–Bauer disk diffusion technique. Anaerobic, fungal, and viral cultures were not done due to the lack of laboratory facilities.

## Infections

For the purposes of this study, we utilised the IDSA criteria [14] for identifying episodes of febrile neutropenia as a single oral temperature measurement of higher than 38.3°C, a temperature of 38°C or higher for longer than one hour, or the occurrence of three temperatures of 38°C or more within a 24-h period, taken at least four hours apart, in a neutropenic patient. Blood cultures were taken the first time the patient had an oral temperature of more than 38.3°C. Throat swabs, swabs from the ear, sputum, and from the skin lesions present were also taken and the organisms identified. The diagnosis of pneumonia was considered if the patient had a suggestive chest x-ray finding associated with equivalent clinical presentation. Septicaemia was defined as the presence of at least one positive blood culture in a patient. Gastrointestinal tract infections included oral infections such as thrush, or significant ulcerations containing pathogenic organisms, dental infections, and enteritis. Urinary tract infection was considered to be present if the patient had a clinical presentation and greater than 10 pus cells in urinalysis. The presence of cellulitis was established based on clinical signs. Documented infections were defined as being either microbiologically or clinically evident, or termed fever of unknown origin when there was no clinical or microbiological documentation but the clinical course was compatible with infection.

A second febrile episode was assigned if a patient developed a new fever after being afebrile for at least 48 h or if new infectious symptoms developed even if the patient had never completely recovered from the febrile episode.

CRP is an acute phase reactant of inflammation, the levels of which can be measured from the serum. In normal healthy individuals and in the absence of infection, the serum levels are usually less than 10 mg/L [15]. Serum CRP levels of more than 40 mg/L have been shown to be suggestive and sensitive markers for bacterial infections in immunocompromised febrile patients [16]. Therefore, we set the value of 40 mg/L as a cutoff point for identification of suspected bacterial infection in our study population.

Hence, the baseline serum levels of CRP in non-febrile patients of leukaemia were measured and compared with the levels after the onset of fever in patients with neutropenia. Their levels were also measured after the institution of antimicrobial agents, and their levels in relation to our cutoff value of 40 mg/L were noted. Measurement of CRP was performed by means of an immunoturbidimetric method in a Fully Automated Random Access Biochemistry Analysis (model ERBA XL-640) using commercially available kits (Transasia, Biomedical Ltd). The CRP level was measured as milligram per decilitre. This value was then converted to milligram per litre.

The treatment regimen used for these febrile neutropenic patients in our unit was as follows:

- Initial therapy: piperacillin-tazobactam combination (4.5 g in 100 ml normal saline 8 hourly) on diagnosis of fever
- Fever persisting after 72 h: linezolid was added
- Fever persisting after 96 h: initial therapy terminated, imipenem + linezolid+ ceftazidime
- Fever persisting after 120 h: intravenous amphotericin B or fluconazole added

Treatment was considered to have been successful if the following were attained without a change in the regimen: the temperature becomes normal and the symptoms and signs of infection at identifiable sites of infection disappear.

The onset of a febrile episode in a neutropenic patient was managed by prompt baseline blood cultures and institution of empirical antibiotics. The antimicrobial therapy was then subsequently tailored per the microorganism isolated, and its antimicrobial susceptibility profile reports and empirically in those not responding after 72 h of treatment. In patients with negative cultures, the empirical antimicrobials were continued until the fever and neutropenia resolved or for a minimum of seven days.

## Data collection and statistical analysis

Data about age at the time of diagnosis, sex, treatment protocol, and other parameters described were collected and compiled for each patient. The day a patient received chemotherapy was defined as day one of that cycle, with the cycle considered as ending upon recovery of ANC to 0.5 × 10^9^/L. The duration of hospital stays and number of febrile days were also measured. The observed infections were divided per the WHO criteria [17].

Univariate results were expressed as medians, ranges, and percentages. Comparison between continuous variables was done using Student’s *t*-test. A *p *value < 0.05 was taken as significant. All statistical analysis was carried out using the software package SPSS, version 13.0 (SPSS, Inc., Chicago, IL, USA).

## Observations and results 

### Demographic profile and characteristics of study population prior to chemotherapy 

The study population consisted of 120 patients, of which 71 (59.8%) were diagnosed to have acute lymphoblastic leukaemia (ALL), while 49 (40.2%) had acute myelogenous leukaemia (AML). All 120 patients were given induction chemotherapy per protocol. Among the 120 patients, 107 patients had neutropenia, while 13 did not have neutropenia. All 107 patients with neutropenia had evidence of infections. Five patients who did not have neutropenia also had infections (from the 13 non-neutropenic subjects). Thus, 112 patients who had evidence of infection had febrile episodes, while eight patients of the non-neutropenic group did not have a fever. Out of the 112 patients with evidence of infection, 67 of them were patients of ALL, while 45 of them were AML patients.

The majority of the patients were of the age group of 26–35 years (31.7%), followed by 25% in the age group of 16–25 years and with a male-to-female ratio of approximately 3:1 as evident by 91 male patients in comparison with 29 female patients (Table 2). The median age of these patients was 28 years, ranging from two to 50 years. In 89.2% of these patients, the absolute neutrophil count was normal and was more than 0.5 × 10^9^/ L prior to chemotherapy as compared with 10.8% of them who had counts less than 0.5 × 10^9^/L. Induction chemotherapy was associated with febrile neutropenic episodes that occurred in a total of 112 patients, which included 67 ALL patients and 45 AML patient.

### Factors influencing infection and outcome in acute leukaemia with neutropenic febrile episodes 

A total of 172 febrile infectious episodes were recorded among 112 patients, out of whom 22 patients had more than one NFEs. Of them, 107 patients were associated with neutropenia, where the absolute count was less than 0.5 × 10^9^/L, while 13 patients did not manifest with neutropenia for their count (although decreased from their pretreatment values) remained within normal limits of more than 0.5 × 10^9^/L. The period between the first day of induction chemotherapy and the onset of neutropenia (range of one to five days) was almost same in both ALL and AML patients. As evident from Table 3, the mean duration of neutropenia was 14 days (range of seven to 25 days) in ALL patients while it was seven days (range of seven to 15 days) in AML patients undergoing induction chemotherapy. The longest duration of neutropenia was observed in acute lymphoblastic leukaemia. The neutropenic infectious febrile episodes in these patients occurred between one to 15 days from the onset of neutropenia to the day on which the patient presented with fever, with a mean of three days. The neutrophil counts on day of onset of fever ranged from 0.05 × 10^9^/L to 0.30 × 10^9^/L with a mean of 0.05 × 10^9^/L. The duration of fever ranged from four hours to 13 days, median being 2.95 days. The mean duration of hospitalisations for AML patients was nine days (range from seven to 15 days), as compared with an average of 10 days in ALL patients (range from five to 35 days).

Among non-neutropenic patients, infection could be documented in 36%; the remaining 64% were designated as isolated febrile episodes. All the neutropenic febrile episodes resolved completely, with a 30-day survival rate of 100% as there were no deaths during any of the febrile neutropenic episodes.

As evident from Table 4, the factors that significantly increased the risk of infection during induction chemotherapy in acute leukaemia were neutropenia (95.5%), presence of an intravenous line (67.9%), presence of any mucosal injury (80.4%) and a history of bone marrow puncture (89.3%), and blood transfusions (56.3%) during the present induction period. The increased use of intravascular devices and mucosal injury made the patients more liable to infections.

### Clinical pattern of neutropenic febrile infections 

Table 5 depicts the total number of documented infections, which was greater than the total number of patients (112 patients with 172 febrile episodes) because of the multiple sites of infection in 10 patients.

Respiratory tract infection accounted for 32.6% of the infections, followed by oral mucosal infections (20.4%), GIT (18.0%), genitourinary tract (6.4%) and skin, and soft tissue (3.5%) infections, in order of decreasing frequency. Other documented infections were otitis media in seven episodes, pneumonia in 10 episodes, and herpes zoster in three, while two patients had pulmonary tuberculosis and one had a discharging sinus from which *Acinetobacter *was the predominant microorganism to be identified.

Among these 172 episodes, an infectious aetiology could be documented in 53 episodes where potential pathogens were cultured (37 organisms from blood and 16 microorganisms from other sites); the remainder was defined as isolated febrile episodes. As evident from Table 6, among the 37 microorganisms isolated from blood cultures, 38.3% were Gram-positive organisms, and 27.7% Gram-negative organisms, while there were four documented fungaemias, contributing to 8.5% of all the microbiologically documented infections. *Escherichia coli*, *Klebsiella*, *Pseudomonas*, *Acinetobacter *and *Salmonella *were the important Gram-negative bacilli isolated, whereas *Staphylococcus aureus *and coagulase-negative staphylococci were the common Gram-positive bacilli isolated. Among them, 4.3% were mixed Gram-negative and Gram-positive infections. Two patients had tuberculosis with multiple positive sputum cultures. Moreover, some patients had grade two to grade four mucositis. There were 10 pneumonias, all of which were diagnosed clinically and by chest x-ray. However, microbial growth was not seen in 10 of the specimens. From the rest of the 16 microorganisms isolated from sites apart from blood, there were five Gram-positive, seven Gram-negative and four mixed Gram-positive and Gram-negative organisms, as evident from Table 7.

### Correlation with serum C-reactive protein levels

As evident from Table 8, on the first day of the induction chemotherapy cycle (baseline), mean CRP values of all the study subjects of CRP was nine mg/L with a range of 0–35 mg/L. On the first day of NFEs, a CRP value of more than 40 mg/L was observed in 32.1%, while a value less than 40 mg/L was seen in 67.9% of patients. In spite of the existing infectious conditions, the serum CRP level beyond the cutoff level of more than 40 mg/L was not seen in 67.9% of the subjects. On the second and third days, 64.3% of the patients with NFEs and 70.6% of them had raised CRP levels of more than 40 mg/L, respectively. This thereby suggests that the significantly higher concentration in CRP levels was seen in the neutropenic febrile patients. Conversely, in 67.9% and 54.5% of the patients, the CRP levels declined from their raised value after three and four days of institution of antimicrobial therapy, respectively, indicating a correlation between the CRP level and the incidence of infection. Hence such findings might indicate the clinical usefulness of CRP levels as a source of diagnostic as well as a prognostic parameter of unapparent infection in critically ill septic cancer patients.

## Management

All the patients were placed on prophylactic empirically antimicrobial therapy. Out of the 172 NFEs, 44 episodes were treated with a combination piperacillin-tazobactam, and 32 (72.7%) responded without modification. Ceftazidime + Meropenem + Linezolid were used in 48 episodes, with a response without modification in 21 episodes (43.8%). Piperacillin + Tazobactam + Amikacin were used in 16 episodes, and six (37.5%) responded without modification. Other varying combinations of antimicrobials were used in 15 episodes. Four of the 15 episodes were treated with acyclovir, while six were treated with amphotericin B. The documented cases of tuberculosis were treated by antitubercular drugs.

## Discussion

Infections in the immunocompromised host as a result of cancer chemotherapy is an important problem in the present day-to-day treatment care, as they are associated with an increased incidence of neutropenic infectious complication, which in turn influences the outcome of the chemotherapeutic response, and thereby morbidity and mortality, in these patients [18]. Thus, prevention and treatment of infection are vital in the management of acute leukaemia, which can be achieved by empirical antibiotic therapy covering the broadest spectrum of organisms because these patients have a 60% likelihood of being infected due to neutropenia [1].

The major focus of this study was on the outcome in febrile neutropenic patients treated in a haematology general ward. In these circumstances, the conditions and equipment are inferior to those in ICUs and the possibilities for respiratory and circulatory support are also limited.

In the present study, the episode of infection was more frequent in subjects with an ANC of <0.5 × 10^9^/L, which suggested that neutropenia increased the risk of infection and sepsis. This goes hand in hand with Pagano, *et al. *who have also found neutropenia as a significant risk factor in similar patients [19].

Bacteraemia and fungaemia could be identified in 37 (21.5%) of the 172 NFEs, where the gram-positive microbes were responsible for the majority of the bloodstream infections. This observation was similar to other reports, where a retrospective, descriptive survey of AML patients who received intensive chemotherapy at the Haematology Units involving 49 hospitals in the USA in 1995 and 2000 also found Gram-positive bacteria to be responsible for 62–76% of the infections, while Gram-negative bacteria accounted for 14% of the bloodstream infections [20]. This is in contrast to other studies where Gram-negative bacilli were found to cause a majority of infections in NFEs [21]. The reasons most likely being that the prophylactic use of antimicrobials in our study, which inhibited the Gram-negative infections leading to a resultant shift to increased Gram-positive bacteraemias. In addition, the judicious use of intravenous lines and a number of chemotherapy-induced mucositis in our study might have increased the frequency of Gram-positive organisms, suggesting that injury to the skin and mucous membranes could have made the patient more liable to infections, caused by the patient’s own endogenous flora. Among the Gram-positive microbes, *S. aureus *was the predominant pathogen to be isolated in febrile neutropenic patients in this study, which is in line with other reported studies [22, 23].

The lungs and upper respiratory airways were the most common sites of localisation of infection, which is also similar to the observations in other studies [24]. On the other hand, other studies have observed the gut and the genitourinary tract to be the most common sites of infections in some NFEs [21]. Some of the reported NFEs showed an increased incidence of associated fungal infections, where such invasive fungaemias were associated with increased morbidity and mortality [25]. Although *Candida albicans *was responsible for 51.8% in neutropenic infections, as reported by other researchers [26], this causative organism accounted for only 8.51% of the total infections in the present study, which was probably due to the prophylactic use of fluconazole in febrile neutropenic patients in our tertiary care.

Studies on neutropenic febrile patients admitted to the ICUs report an increased incidence of hospital-acquired infections like ventilator-associated pneumonia, congestive cardiac failure and intravascular catheter infections, which were predominantly caused by Gram-positive organisms, especially the coagulase-negative *S. aureus *[27]. Use of indwelling catheters, femoral insertion sites, and the choice of skin antisepsis were some of the reported modifiable risk factors for infection in an ICU [28]. Some of these admitted infected neutropenic patients had a significantly longer duration of hospital stay consequent to higher rates of clinical sepsis and a resultant increase in crude mortality rates [29].

The usefulness of the serum levels of CRP in the early diagnosis and in the monitoring of the course of bacterial infections in febrile neutropenic patients with cancer was also evaluated in the present study. A majority of the febrile neutropenic patients with bacterial infections had significantly higher serum CRP levels. A decline in the serum CRP levels was observed in patients who had a favourable response to antimicrobial treatment, which suggests that serum CRP levels may indicate the occurrence and prognosis of bacterial infection and therefore can aid in identification of neutropenic patients who are at high risk for bacterial infections. Thus, as evident from this study, the usefulness of serial determinations of serum CRP for the diagnosis and follow-up of bacterial infections can be relied upon, although its usefulness in the evaluation of neutropenic febrile patients with cancer has always been debated upon by many researchers, for it has been postulated that serum CRP levels can also be elevated as a consequence of the malignant process itself, irrespective of the presence of systemic bacterial infections [30].

As covered by the guideline scope, the two methods of prophylaxis against neutropenic sepsis are antibiotics and G-CSF [31]. Yet in contrast to such existing guidelines, it was observed that although such growth factors (G-CSF) were never used during the patient care protocol in our study, there was still a favourable outcome. Thus, they may not really be necessary and they should be used stringently and in patients who can afford their exorbitant costs.

The majority of the infectious episodes in our patients (124 episodes from 172 infectious episodes) were treated initially with a combination of antibiotic therapy consisting of an antipseudomonal antibiotic with either aminoglycoside or glycopeptides or a β-lactam antibiotic, with a favourable response rate. The rest of the episodes was managed with varied combinations of differing antimicrobials. Although various empiric regimens have been recommended for febrile neutropenic patients, it is, however, difficult to adopt a single recommended regimen, as not only the spectrum of bacterial isolates varies from one setting to another but also the results of various studies are at times not comparable because of differing criteria [32]. Traditionally, a combination therapy of an aminoglycoside with an anti-pseudomonal β-lactam is preferred in many setups [32]. Carbapenems and piperacillin-tazobactam are also being increasingly used [32, 33]. On the other hand, some studies have emphasised a role for high-dose quinolones such as ciprofloxacin as a monotherapy in neutropenic patients [34], while others have recommended newer quinolones such as clinafloxacin [35].

Prophylactic antibiotic therapy is currently being practiced in the Clinical Hematology Leukaemia Unit of our centre for patients with acute leukaemia undergoing intensive chemotherapy, so the rate of NFEs in our unit was not high (112 patients with 172 infectious episodes); they could be managed effectively without any mortality, and prophylactic measures improved the outcome of infection in neutropenic patients. Other studies that have doubted the value of prophylactic antibiotics in neutropenic patients, having reports of 14 patients presenting with 43 episodes of NFE [36].

However, emergence of antibiotic resistance and super infection needs careful consideration. Besides the fact that the value of such prophylactic antibiotics in neutropenic patients is controversial and needs to be reconsidered [37], some authorities recommend giving prophylactic cotrimoxazole to only granulocytopenic children [38].

Although the prevention of Gram-positive infections is \ more difficult to treat, co-trimoxazole and penicillin have been used and found to be effective in some studies [39]. Prophylaxis of fungal and viral infection is another important issue, but in certain studies it was largely limited to the patients who underwent an allogeneic bone marrow transplant [40].

In the patients in the present study, fever without any obvious infectious source also responded to antimicrobial therapy, suggesting that most febrile episodes were infectious. A median of five days between the onset of chemotherapy and the manifestations of NFEs was seen, which was slightly shorter as compared with previous reports in the literature [29, 30]. A 100%, 30-day survival rate was observed in our study, for none of the NFEs resulted in a fatal outcome. This is in contrast to other studies where the observations suggest that the patients with active leukaemia have an increased risk of death [41]. The results of the FINNSEPSIS study also reveal that only 86% of the patients admitted for sepsis to ICU recovered, while the rest succumbed [42].

The ICU length of stay (43 days) for patients with infection and ventilator-associated pneumonia was also considerably longer than for patients with one infection (19 days) or no infection (four days) [27].

Our study thus reports that acute leukaemia patients on chemotherapy with fever and neutropenia, although at risk of bacteraemia/fungaemia, could be managed safely in general wards with favourable outcome and early hospital discharge. This is in accordance with other studies that state such patients can be managed effectively outside an ICU setting [43]. Some authors have also estimated that this approach could save $5,000 per patient (United States dollars) and $23,000,000 per year for the United States [44]. In a pilot study, this same group has also successfully treated 19 children at low risk for bacteraemia without the aid of an ICU [45].

We assume that not all patients with neutropenic sepsis are at the same risk of developing severe sepsis and that the treatment and location of treatment may be tailored according to the risk factors and other circumstances. Most patients also prefer to be treated at home where the risks of hospital-acquired infections are lower, along with potential reduced cost, leading to the saving of limited resources.

## Conclusion

Sepsis is a serious yet common complication in cancer patients on chemotherapy, as the consequent neutropenia and granulocytopenia may result in significant morbidity and mortality. Although great progress has been made in this field, the management of febrile neutropenic patients still remains a challenge. This study reveals that the use of early empirical antibiotic therapy and maintenance of proper hygiene is a reasonable, effective, and successful way to combat infections, complications, and death in general wards. Acute leukaemia can thus be managed effectively in resource-poor centres with optimum utilisation of resources, if referral to specialised centres is not feasible.

## Limitations of the study

It may also be possible that some of the microorganisms were missed in blood cultures or not detected due to lack of facilities for anaerobic and viral cultures.

## Figures and Tables

**Table 1:**
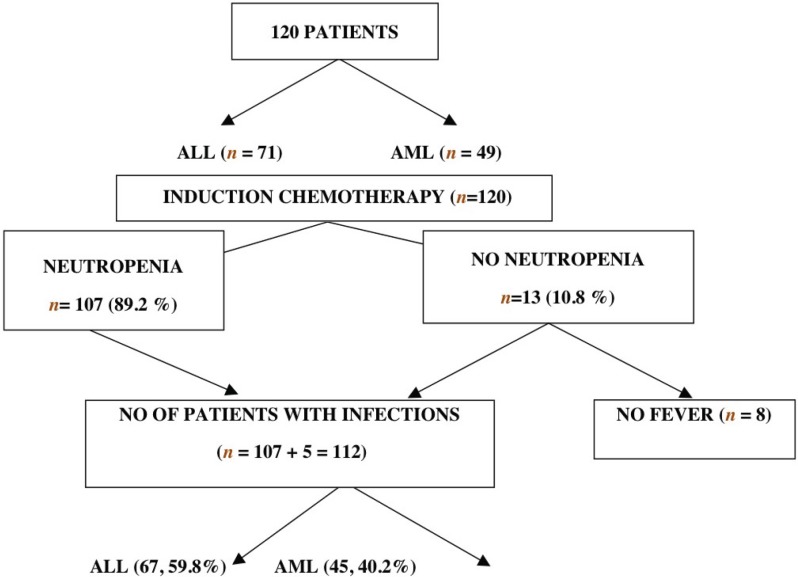
Study protocol for acute leukaemia patients during febrile neutropenic episodes

**Table 2.** Characteristics of patients prior to chemotherapy (*n*=120)

Parameters		ALL (*n* = 71)	AML (*n* = 49)	Total (*n* = 120)
**Age (years)**	2–16	16	10	26 (21.7%)
	16–25	18	12	30 (25%)
	26–35	22	16	38 (31.7%)
	36–50	15	11	26 (21.7%)
**Sex**	Male	55	36	91 (75.9%)
	Female	16	13	29 (24.2%)
**Haemoglobin**	< 8 g/dl	18	31	49 (40.9%)
	≥ 8 g/dl l	53	18	71 (59.2%)
**Absolute neutrophil count**	< 0.5 × 10^9^/L	8	5	13 (10.9%)
	≥ 0.5 × 10^9^/L	63	44	107 (89.2%)
**Platelet count (per cubic millimetre)**	< 10,000	11	7	17 (14.2%)
	> 10,000	60	42	102 (85%)

**Table 3.** Factors influencing infection and outcome

Parameters	ALL	AML
**No of patients on induction courses**	71	49
**Mean days of neutropenia (range)**	14 (7-25)	7 (7-15)
**Mean days from chemotherapy to onset of neutropenia**	5	
**Mean days from onset of neutropenia to fever**	3 (1-15)	
**No of neutropenic episodes**	107	
**Total no of febrile episodes**	172	
**No of patients with more than 1 febrile episode**	22	
**Duration of fever**	4 h-13 days	
**Absolute neutrophil count at the onset of fever**	0.05 (0.05-0.30) 10^9^/L	
**No of patient with mucositis of grade 2-4 (55)**	31	24
**Clinically documented infections (172)**	10	12
**Microbiologically documented infection (37+16)**	22	31
**Mean days of hospitalisation (range)**	10 (5-35)	9 (7-15)

**Table 4.** Factors affecting risk of infection in acute leukaemia (*n*=112)

Risk factors		Infections	*p*
**Neutropenia**	Yes	107 (95.5%)	<0.05
	No	5 (4.5%)	
**Presence of intravenous line**	Yes	76 (67.9%)	<0.05
	No	36 (32.1%)	
**Mucosal barrier injury**	Yes	90 (80.4%)	<0.05
	No	22 (19.6%)	
**History of bone marrow puncture**	Yes	100 (89.3%)	<0.05
	No	12 (10.7%)	
**History of blood transfusion**	Yes	63 (56.3%)	<0.05
	No	49 (43.8%)	
**Use of corticosteroids**	Yes	All patients	
	No	---	

**Table 5.** Clinical pattern of neutropenic febrile infections (112 patients with 172 infectious episodes)

Risk factors	Infections	*p*
**Respiratory tract infection**	56	32.6
**Mucositis/gingivitis/oral ulcers**	35	20.4
**Gastrointestinal infection**	31	18.0
**Genitourinary infection**	11	6.4
**Pneumonia**	10	5.9
**Otitis/mastoiditis**	7	4.1
**Mixed infections**	7	4.1
**Skin and soft tissue infections**	6	3.5
**Cellulitis**	3	1.7
**Herpes zoster**	3	1.7
**Tuberculosis**	2	1.6
**Discharging sinus**	1	0.6
**Total no of infectious episodes**	172	100
**Multiple sites of infection**	10	

**Table 6.** Microorganisms isolated from blood cultures

Organisms isolated		Number	%
**Gram positive 38.30%**
	***Staphylococcuscoagulase positive***	8	17.0
	***Staphylococcuscoagulase negative***	6	12.8
	***Bacillus subtilis***	2	4.3
	***Streptococcus pneumoniae***	1	2.1
	***Streptococcus sp.***	1	2.1
**Gram negative 27.66%**
	***Escherichia coli***	4	8.5
	***Acinetobacter***	3	6.4
	***Enterobacter***	3	6.4
	***Pseudomonas***	1	2.1
	***Salmonella***	1	2.1
	***Klebsiella***	1	2.1
**Fungal**	***Candida* sp.**	4	8.5
**Mixed gram positive and gram negative**		2	4.3
**Total organisms isolated**		37	78.7
**Non-growth**		10	21.3
**Total samples collected**		47	100

**Table 7.** Microorganisms isolated from other sites

Organisms isolated		Sites	Number
**Gram positive (5)**	***Streptococcus***	Throat	2
	***Bacillus subtilis***	Sinus	1
	***Mycobacteria***	Sputum	2
**Gram negative 27.66%**	***Escherichia coli***	Stool	3
	***Pseudomonas***	Urine	1
		Ear	1
	***Klebsiella***	Urine	2
**Mixed (4)**		Urine	3
		Stool	1
**Total organisms isolated**			16

**Table 8.** Correlation between serum CRP (mg/L) and NFEs

Days of measurement	CRP levels (mg/L)	No of patients (%)
**Day 1 of Chemotherapy cycle**	Less than 10 (0-35)	112 (100%)
**Day 1 of NFEs**	More than 40	36 (32.1%)
	Less than 40	76 (67.9%)
**Day 2 of NFEs**	More than 40	72 (64.3%)
	Less than 40	40 (35.7%)
**Day 3 of NFEs**	More than 40	79 (70.6%)
	Less than 40	33 (29.5%)
**Antimicrobials in NFEs (day 4 of NFEs)**	More than 40	42 (37.5%)
	Less than 40	70 (67.9%)
**Antimicrobials in NFEs (day 7 of NFEs)**	More than 40	51 (45.6%)
		61 (54.5%)
